# Regarding: A Common Source Outbreak of Anisakidosis in the United States and Postexposure Prophylaxis of Family Collaterals

**DOI:** 10.4269/ajtmh.18-1019

**Published:** 2019-03

**Authors:** Sarah G. H. Sapp, Richard S. Bradbury, Henry S. Bishop, Susan P. Montgomery

**Affiliations:** Parasitic Diseases Branch; Division of Parasitic Diseases and Malaria; Centers for Disease Control and Prevention; Atlanta, Georgia

Dear Sir,

The case recently reported by Carlin et al.^1^ was brought to our attention, and we wish to address some details regarding the morphologic diagnosis of anisakiasis. Whereas the histological section of the patient’s small bowel biopsy shown in Figure 1 illustrates an example of an *Anisakis* sp. larva with clearly evident diagnostic features (particularly, the size in relation to surrounding tissue, tall polymyarian and coelomyarian musculature, and prominent Y-shaped lateral chords) that provide sufficient evidence for the diagnosis,^2^ Figure 2 features an object that does not appear to be a helminth. The morphologic criteria used to identify the object as *Anisakis* sp. are not specified, nor are any such features made visible to the reader. The typical appearance of anisakid larvae recovered from human infections is of a loosely coiled, white to pinkish larva, usually 2–3 cm long with three lips, a boring tooth, and a mucron (small projection) on the posterior extremity. Further generic diagnosis is based on the morphology of the esophageal–intestinal junction and lateral chords.^3^

Artifacts such as epithelial casts, mucus strands, and undigested food that may be coincidentally passed after treatment are commonly mistaken as helminthic parasites. Notably, artifacts identified as “rope worms” are an example of such misidentifications. Passage of anisakid larvae in stools is not well documented in the literature, and we have not encountered this phenomenon in our prior experience as a parasitic disease reference diagnostic laboratory.

Morphological diagnosis of parasites is a niche discipline that requires specialized training and extensive experience. The Parasitic Diseases Branch at the Centers for Disease Control and Prevention offers diagnostic assistance through DPDx (Centers for Disease Control and Prevention, Atlanta, GA),^4^ a web resource dedicated to the laboratory identification of human parasites. Telediagnosis based on images, case consultation, and direct examination of specimens are offered free of charge. We highly encourage clinicians, pathologists, and laboratory scientists who do not have extensive parasitic morphology training to use the services of parasitic diseases reference laboratories and ancillary resources such as DPDx for guidance in the diagnosis of parasitic diseases.
